# Strategies, processes, outcomes, and costs of implementing experience sampling-based monitoring in routine mental health care in four European countries: study protocol for the IMMERSE effectiveness-implementation study

**DOI:** 10.1186/s12888-024-05839-4

**Published:** 2024-06-24

**Authors:** Ulrich Reininghaus, Matthias Schwannauer, Islay Barne, Joanne R. Beames, Rafaël A. Bonnier, Manuel Brenner, Dagmar Breznoščáková, Daniel Dančík, Manuela De Allegri, Simona Di Folco, Daniel Durstewitz, Jessica Gugel, Michal Hajdúk, Anton Heretik, Ľubomíra Izáková, Zuzana Katreniakova, Glenn Kiekens, Georgia Koppe, Adam Kurilla, Luca Marelli, Iveta Nagyova, Hoa Nguyen, Ján Pečeňák, Julia C. C. Schulte-Strathaus, Koraima Sotomayor-Enriquez, Lotte Uyttebroek, Jeroen Weermeijer, Maria Wolters, Michel Wensing, Jan R. Boehnke, Inez Myin-Germeys, Anita Schick

**Affiliations:** 1 Department of Public Mental Health, Central Institute of Mental Health, Medical Faculty Mannheim, Heidelberg University, Mannheim, Germany; 2 Centre for Epidemiology and Public Health, Health Service and Population Research Department, Institute of Psychiatry, Psychology & Neuroscience, King’s College London, London, UK; 3 German Center for Mental Health (DZPG), partner site Mannheim-Heidelberg-Ulm, Mannheim, Germany; 4 School of Health in Social Science, University of Edinburgh, Edinburgh, UK; 5 Center for Contextual Psychiatry, Department of Neurosciences, KU Leuven, Leuven, Belgium; 6 Hector Institute for AI in Psychiatry, Central Institute of Mental Health, Medical Faculty Mannheim, Heidelberg University, Mannheim, Germany; 7 Department of Psychology, Faculty of Arts, Comenius University, Bratislava, Slovakia; 8 Department of Psychiatry, Faculty of Medicine, Comenius University, Bratislava, Slovakia; 9 Heidelberg Institute of Global Health, University Hospital and Faculty of Medicine, Heidelberg University, Heidelberg, Germany; 10 Department of Theoretical Neuroscience, Central Institute of Mental Health, Medical Faculty Mannheim, Heidelberg University, Mannheim, Germany; 11 Department of Psychiatry and Psychotherapy, Central Institute of Mental Health, Medical Faculty Mannheim, Heidelberg University, Mannheim, Germany; 12 Department of Social and Behavioural Medicine, Faculty of Medicine, PJ Safarik University, Kosice, Slovakia; 13 Faculty of Psychology and Educational Sciences, Clinical Psychology, KU Leuven, Leuven, Belgium; 14 Department of Medical and Clinical Psychology, Tilburg University, Tilburg, The Netherlands; 15 Centre for Sociological Research, KU Leuven, Leuven, Belgium; 16 Department of Medical Biotechnology and Translational Medicine, University of Milan, Milan, Italy; 17 School of Informatics, University of Edinburgh, Edinburgh, UK; 18 OFFIS Institute for Information Technology, Oldenburg, Germany; 19 Department of General Practice and Health Services Research, Heidelberg University Hospital, Heidelberg, Germany; 20 School of Health Sciences, University of Dundee, Dundee, UK

**Keywords:** mHealth, Experience Sampling Method, Ecological Momentary Assessment

## Abstract

**Background:**

Recent years have seen a growing interest in the use of digital tools for delivering person-centred mental health care. Experience Sampling Methodology (ESM), a structured diary technique for capturing moment-to-moment variation in experience and behaviour in service users’ daily life, reflects a particularly promising avenue for implementing a person-centred approach. While there is evidence on the effectiveness of ESM-based monitoring, uptake in routine mental health care remains limited. The overarching aim of this hybrid effectiveness-implementation study is to investigate, in detail, reach, effectiveness, adoption, implementation, and maintenance as well as contextual factors, processes, and costs of implementing ESM-based monitoring, reporting, and feedback into routine mental health care in four European countries (i.e., Belgium, Germany, Scotland, Slovakia).

**Methods:**

In this hybrid effectiveness-implementation study, a parallel-group, assessor-blind, multi-centre cluster randomized controlled trial (cRCT) will be conducted, combined with a process and economic evaluation. In the cRCT, 24 clinical units (as the cluster and unit of randomization) at eight sites in four European countries will be randomly allocated using an unbalanced 2:1 ratio to one of two conditions: (a) the experimental condition, in which participants receive a Digital Mobile Mental Health intervention (DMMH) and other implementation strategies in addition to treatment as usual (TAU) or (b) the control condition, in which service users are provided with TAU. Outcome data in service users and clinicians will be collected at four time points: at baseline (t_0_), 2-month post-baseline (t_1_), 6-month post-baseline (t_2_), and 12-month post-baseline (t_3_). The primary outcome will be patient-reported service engagement assessed with the service attachment questionnaire at 2-month post-baseline. The process and economic evaluation will provide in-depth insights into in-vivo context-mechanism-outcome configurations and economic costs of the DMMH and other implementation strategies in routine care, respectively.

**Discussion:**

If this trial provides evidence on reach, effectiveness, adoption, implementation and maintenance of implementing ESM-based monitoring, reporting, and feedback, it will form the basis for establishing its public health impact and has significant potential to bridge the research-to-practice gap and contribute to swifter ecological translation of digital innovations to real-world delivery in routine mental health care.

**Trial registration:**

ISRCTN15109760 (ISRCTN registry, date: 03/08/2022).

**Supplementary Information:**

The online version contains supplementary material available at 10.1186/s12888-024-05839-4.

## Contributions to the literature

- The present hybrid effectiveness-implementation study will improve our understanding of strategies, contextual factors, processes, outcomes, and costs of implementing ESM-based monitoring, reporting and feedback into routine mental health care.
- The DMMH reflects a step change in regulatory-compliant delivery of ESM-based monitoring, reporting and feedback as a technology-enabled service, facilitated by technological, user- and stakeholder-centred implementation strategies.
- The study will advance true person-centred care, empowering service users as active partners in treatment, self-management, and decision-making.
- Coupled with the work carried out in the wider EU IMMERSE consortium, the study will contribute to the digital transformation of mental health care in Europe.

## Background

Recent years have seen a growing interest in the use of digital tools for delivering person-centred mental health care [1, 2]. Experience Sampling Methodology (ESM) is a structured diary technique for collecting intensive longitudinal data on moment-to-moment variation in experience and behaviour in service users’ daily life and, as such, identifies them as experts of their own experience [1–3]. ESM therefore reflects a particularly promising avenue for adopting a person-centred approach through digital monitoring, reporting, and feedback in routine mental health care [1, 2, 4]. Such an ESM-based, person-centred approach may strengthen service user engagement and empowerment by making service users active partners in, rather than passive recipients of their own treatment and care [1, 2, 4]. It may further improve self-management and recovery, as it provides service users with a tool to enhance their understanding of, and better manage their own mental health problems [5, 6]. The ESM-based, person-centred approach may also provide goal direction in clinical assessment and management of care, as the detailed information on patterns of variation in, co-variation of symptoms, key problem areas and relevant contexts will help clinicians and service users to set clear, actionable and personalized therapy goals [4]. Finally, this approach may enhance shared decision making, as it provides strongly needed and relevant day-to-day information on key problem areas and relevant contextual factors as a basis for jointly making treatment decisions and evaluating progress of treatment [4].

While there is evidence on the effectiveness of ESM-based monitoring, reporting, and feedback in people with mental health problems [5, 7–9], uptake remains limited due to a lack of digital tools that comply with regulatory requirements and, hence, would allow for ESM-based monitoring, reporting, and feedback in routine care settings [2, 10–12]. Several regulatory, technological, clinical and organizational implementation barriers need to be addressed and overcome to realize more fully its potential through developing and evaluating strategies for effective implementation in mental health care practice [10, 12, 13]. In fact, research on the implementation of mHealth tools in routine mental health care in general remains very limited [12], which may explain the marked research-to-practice gap [10, 11]. The current hybrid effectiveness-implementation study [14] sets out to directly address this challenge as part of the EU Horizon 2020 funded Implementing Mobile MEntal health Recording Strategy for Europe (IMMERSE) consortium.

The overarching aim of this hybrid effectiveness-implementation study is to investigate, in detail, strategies, contextual factors, processes, outcomes, and costs of implementing ESM-based monitoring, reporting, and feedback into routine mental health care through a Digital Mobile Mental Health intervention (DMMH) and other implementation strategies to facilitate its use in four European countries (i.e., Belgium, Germany, Scotland, Slovak Republic). To this end, a pragmatic parallel-group cluster randomized controlled trial (cRCT) will be conducted and combined with a process and economic evaluation. The DMMH as the core strategy for implementing ESM-based monitoring, reporting, and feedback consists of (1) the MoMent App [2, 15], a digital application for mobile devices using ESM to systematically monitor service users’ self-reported momentary key complaints, symptoms, mood, activities, context, and treatment goals in daily life; and (2) the MoMent Management Console that allows clinicians to (a) personalize treatment goals and questionnaires that are presented by the MoMent App jointly with the individual service user, and (b) generate reports that provide meaningful information from the self-report data using the integrated MoMent Dashboard, an interface to visualize and distil the collected data into tailored feedback to the service users and their clinicians. Implementation strategies will further include technological strategies, strategies for clinicians and service users, and organisational strategies to facilitate the use of ESM-based monitoring, reporting and feedback in routine care via the DMMH. These will be standardized with some variation and flexibility in their application across sites.

To achieve our overarching aim, this study will:

1. Establish, in a pragmatic cRCT of the DMMH and other implementation strategies to support its use in service users and clinicians in routine care settings, i) **R**each (i.e., service user participation), ii) **E**ffectiveness (defined as the interaction of efficacy and implementation in real-world settings), operationalized as greater service user engagement at 2-month post-baseline in the experimental than control condition as primary outcome, iii) **A**doption (i.e., proportion of service users and clinicians having used DMMH components in routine care), iv) **I**mplementation (defined as delivery of the DMMH as intended in routine care) and v) **M**aintenance (defined as the extent to which the DMMH becomes sustainable part of routine care at 6-month and 12-month post-baseline). Consistent with the **RE-AIM** framework [16], this will provide the basis for assessing the public health impact of implementation and scale-up of ESM-based monitoring, reporting and feedback via the DMMH and other implementation strategies. The primary hypothesis will be that, compared with the control condition (i.e., Treatment-As-Usual (TAU)), patient-reported service engagement, assessed with the total score of the Service Attachment Questionnaire (SAQ) at 2-month post-baseline (primary outcome), will be higher in the experimental condition (DMMH + additional implementation strategies + TAU), while controlling for service user engagement and clinical unit at baseline (please see our preregistration published on the Open Science Framework (OSF) [15] for further details on all hypotheses).
2. Understand how service users leverage DMMH to support their health and well-being, and evaluate the process of implementing ESM-based monitoring, reporting and feedback in routine clinical care pathways using a realist evaluation framework [17] to identify in vivo configurations of contexts, processes and mechanisms of action, and how these are associated with outcomes of implementation in service users, clinicians, and managers/system administrators.
3. Investigate the economic costs of the DMMH and other implementation strategies, and determine their cost-effectiveness and cost-utility vis à vis TAU.

## Methods

### Study design

In this hybrid effectiveness-implementation study [14], a parallel-group, assessor-blind, multi-centre cRCT will be conducted, in which 24 clinical units (as the unit of randomization) at eight sites in four European countries are randomly allocated using an unbalanced 2:1 ratio to one of two conditions: (a) the experimental condition, in which participants receive the DMMH and other implementation strategies in addition to treatment as usual (TAU) or (b) the control condition, in which participants are provided with TAU. Outcome data in service users and clinicians will be collected by assessors masked to random allocation of clinical units at four time points: at baseline (t_0_), 2-month (t_1_), 6-month (t_2_) and 12-month post-baseline (t_3_). The cRCT will follow Consolidated Standards of Reporting Trials (CONSORT) reporting guidelines [18] and relevant extensions [19, 20]. Standard Protocol Items: Recommendations for Interventional Trials (SPIRIT) [21] and Template for Intervention Description and Replication (TIDiER) [22] checklists are available as Supplementary Materials 7 and 8. The primary outcome will be patient-reported service engagement assessed with the SAQ at 2-month post-baseline, a measure of service users’ experience of, and engagement with, their treatment and service [23]. The cRCT will be combined with a process and economic evaluation to provide in-depth insights into in-vivo context-mechanism-outcome configurations as well as economic costs of implementing the DMMH in routine care.

### Study population and clusters

**Fig. 1:**
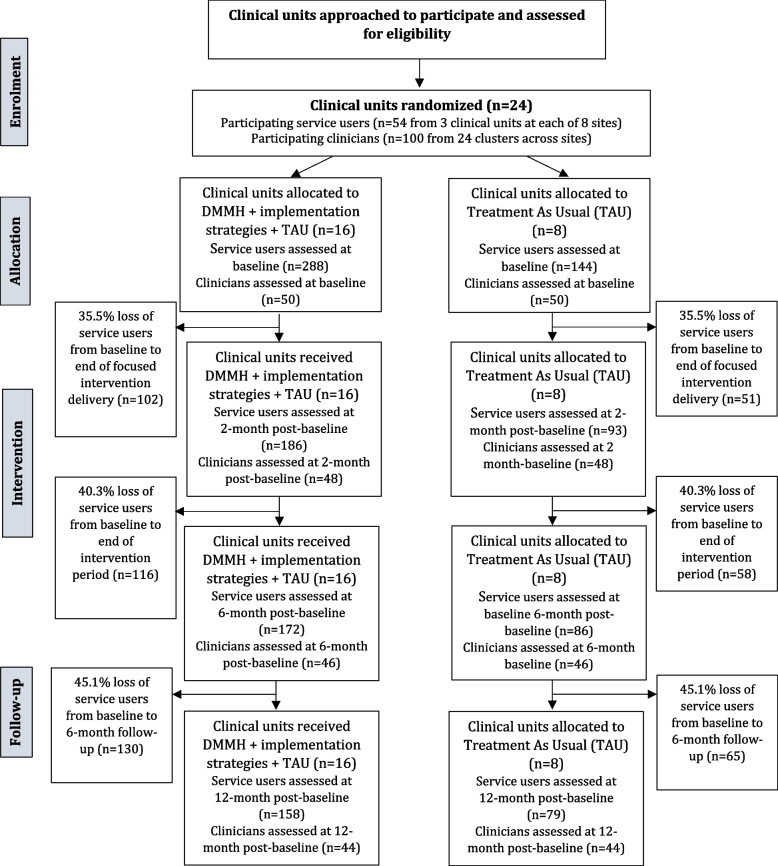
Flow chart

Service users and clinicians comprise the study population in the cRCT, process and economic evaluation in the four European countries, which will be recruited from three clinical units (clusters) in each of the eight clinical sites and, thus, a total of 24 units (clusters). Due to differences in mental health care systems across the four European countries, clinical units (clusters) will vary in structure, service type, and size, which will enhance generalizability of findings and allow implementation to be examined under various conditions. Clinical units (as cluster and unit of randomization) will be community mental health teams (Scotland), tracks (Germany (at the Central Institute of Mental Health Mannheim (CIMH)), inpatient, outpatient and community-based services (Germany (at Psychiatric Centre Nordbaden), Belgium, and Slovak Republic). A clinical unit typically will comprise a multidisciplinary team (i.e., psychiatrists, psychologists, nurses, social workers, occupational therapists). In each of the eight clinical sites, 54 service users will be recruited from three clinical units (i.e., 18 service users per unit), and thus a total of 432 service users (*n*=288 in the experimental condition, *n* =144 in the control condition). In addition, around 100 clinicians of these service users from 24 clusters across all sites are estimated to be recruited in a period of 6 months (see Fig. 1). Recruitment and consent procedures as well as eligibility criteria are described in more detail in Supplementary Material 2.

### Experimental and control condition

#### Control condition: treatment as usual (TAU)

Participants in the control condition will be provided with TAU (i.e., continue to receive all the treatment they received prior to the start of the study). This will include good standard care delivered according to local and national service guidelines and protocols by their general practitioner, psychiatrist and other mental health professionals. Service contacts will be assessed for the duration of the trial using the Client Service Receipt Inventory (CSRI) [24] to monitor variation in delivery of, and engagement with, mental health services and digital technology.

#### Experimental condition: DMMH + additional implementation strategies + TAU

The DMMH and additional implementation strategies supporting its use will be provided in clinical units allocated to the experimental condition in addition to TAU. The DMMH reflects the core strategy for implementing ESM-based monitoring, reporting, and feedback in routine care and consists of (1) the MoMent App and (2) the MoMent Management Console (see above), which are hosted on the Therapy Designer platform by the movisens GmbH (Karlsruhe, Germany). In addition, technological implementation strategies, implementation strategies for clinicians and service users, and organisational implementation strategies will be delivered to facilitate the use of ESM-based monitoring, reporting and feedback in routine care via the DMMH. Both DMMH and additional implementation strategies are described in detail in Supplementary Materials 3 and 7.

The DMMH and additional implementation strategies will be delivered over a 6-month period, including an initial 2-month period for focused delivery of DMMH and implementation strategies, in which service users will be asked to use ESM-based monitoring via the MoMent App, and both service users and clinicians will be provided with detailed numeric and visual reporting and feedback via the MoMent Dashboard (in addition to basic visual feedback via the MoMent App) for at least four weeks. In the remainder of this 6-month period, service users and clinicians will continue to have access to the DMMH and additional implementation strategies. After the end of this period, there will be a 6-month maintenance period, in which service users and clinicians still have access to the DMMH but additional implementation strategies for service users and clinicians requiring active support by the research team will be discontinued.

### Outcome measures

Following the **RE-AIM** framework [16], the evaluation in this hybrid effectiveness-implementation trial will focus on a range of outcomes relating to **R**each, **E**ffectiveness, **A**doption, **I**mplementation, and **M**aintenance of the DMMH, covering service user experience, implementation outcomes, and mental health outcomes. The primary outcome has been selected for investigating the **E**ffectiveness of implementing the DMMH. The primary outcome will be patient-reported service engagement assessed with the SAQ total score [23] at 2-month post-baseline, a measure of service users’ experience of, and engagement with, their treatment and service. It captures a proximal effect of DMMH implementation on service users’ interaction with mental health services and, hence, reflects a primary indicator of implementation success. All primary and secondary outcomes will be assessed at baseline (t_0_), 2-month (t_1_), 6-month (t_2_) and 12-month post-baseline (t_3_). Secondary outcome data collected using ESM will follow the protocol from previous experience sampling studies [1]. Please see Supplementary Material 1 (based on SPIRIT statement) and Supplementary Material 4 for details of all measures used to examine reach, effectiveness, adoption, implementation, and maintenance. The assessment of safety based on the EU Medical Device Regulation (MDR 2017/745) is described in Supplementary Material 5.

### Process evaluation

A process evaluation will be performed using semi-structured interviews with service users, clinicians and managers/system administrators during or at the end of the initial 6-month period. The interviews of the process evaluation will be semi-structured and take a realist evaluation approach [17], which will be combined with the Non-adoption, Abandonment, Scale-up, Spread, and Sustainability (NASSS) framework [25] to establish what works, for whom, in what circumstances, in what respects, to what extent, and why. This implies that configurations of contextual factors, mechanisms of implementation, and outcomes of the implementation and intervention are explored across all levels of agents within the intervention and its implementation (i.e., individual participants, clinicians, managers and system administrators, and socio-economic and contextual factors that may impact their intentionality, behaviour and decision making at different stages of the intervention). This will also allow us to examine how service users and clinicians appropriate DMMH to serve their particular needs. Initial programme theories will be developed based on initial semi-structured interviews. Overarching programme theory and accompanying context-mechanism-outcome configurations will be tested among intervention users (individual interviews with participants who have completed the DMMH) as well as those who deliver the intervention (i.e., clinicians) and providing the context of intervention delivery (i.e., managers/system administrators), through iterative data collection. We will also explore unexpected consequences (positive or negative) on service users and health care professionals, such as impacts on clinical teams and organizations. Taken together, this will allow us to identify key aspects of successful and effective implementation of the DMMH in routine clinical pathways and treatment settings.

### Randomization and blinding

A validated and concealed procedure for randomization will be applied independently of the research team using an unbalanced 2:1 ratio for allocating clinical units to the experimental and control condition stratified by the eight clinical sites without contamination (cross-exposure to the experimental condition). An unbalanced allocation ratio of 2:1 will be used to allow for more detailed investigation of implementation aspects and protect against attrition. This will include an option in the concealed randomization procedure to allocate additional clinical units at each of the eight clinical sites if recruitment rates are lower than expected for some clinical units.

After random allocation of clinical units, clinicians in the experimental condition will be informed about allocation status. This will be done through an independent researcher and not the outcome assessors, who will be blind to allocation status for assessments at baseline, 2-month, 6-month and 12-month post-baseline. Further, there will be an independent contact person, who will not be involved in any assessments, for any questions regarding the recruitment and assessment procedure by service users and clinicians. The trial cannot be fully “blind” because clinicians and service users cannot be masked towards the allocation of clinical units to the experimental or control condition. However, outcome assessors will be blinded to allocation status when assessing eligibility, baseline scores and outcomes at baseline, 2-month, 6-month and 12-month post-baseline. Any data specific to the experimental condition (e.g., on clinical feasibility) will be stored in a separate database. Any breaks in masking will be documented in the trial master file and another blinded assessor will be allocated to repeat the assessment and complete the next set of assessments where possible. To maintain the overall quality and legitimacy of the trial, code breaks will only be done in exceptional circumstances when knowledge of the treatment allocation is absolutely essential for further management of the service user.

Given outcome data will be collected using ESM at baseline, 2-month, 6-month and 12-month post-baseline, and ESM forms a key part of the DMMH (as the experimental manipulation of the cRCT), we will control for any potential confounding of ESM *outcome* data collection by randomly allocating service users to either participation in collecting outcome data (on momentary quality of life, social functioning, and mental ill-health) using ESM or no participation in ESM outcome data collection. This secondary randomization will use a 1:1 ratio in a validated and concealed procedure that will be applied independently of the research team. Notably, this randomization does *not* address any of our study aims on reach, effectiveness, adoption, implementation, and maintenance but will control for (and investigate in sensitivity analyses) the potential effect of ESM data collection (by including a covariate on random allocation to participation in ESM outcome data collection in our statistical models for testing hypotheses on primary and secondary outcomes). The period of additional ESM data collection is independent of the use of the DMMH and other implementation strategies (as the experimental condition of the cRCT). Please see Supplementary Material 4 for further detail.

### Sample size calculation

We will test the primary hypothesis of the effect of the experimental vs. control condition (i.e., DMMH + additional implementation strategies + TAU vs. TAU) on service engagement as primary outcome (measured with the patient-reported SAQ total score as the dependent variable). We will use a fixed effects regression model controlling for unit effects, with a dummy variable for the condition (DMMH + additional implementation strategies + TAU vs. TAU), the SAQ engagement total score at baseline, and a dummy variable coding for the random allocation to ESM data collection at baseline. Ignoring in a first step the correlations between values taken from the same cluster, the total number of service users required to detect an effect of size d=0.4 (i.e., an effect size slightly lower than reported for service engagement in a previous study investigating the effects of an App-based mobile mental health solution [26]), with power 1- β = 0.80 at an alpha level of 0.05 with sample size ratio 2 (experimental condition) : 1 (control condition) is computed to be N0 = 201 for the null hypothesis that the difference between both experimental and control condition in terms of the mean SAQ score is zero at 2-month (t_1_) post-baseline, versus the alternative hypothesis that there is a difference (non-directional alternative). The statistical test will employ a fixed effects linear regression model with a variable representing treatment vs. control as the focal coefficient. The coefficient test will be performed at significance level α = 0.05, with the SAQ score at baseline as control variable and controlling for unit clustering. If all 24 units to be randomized have size n0 = (201/24=) 8.38 and assuming an intraclass coefficient of ICC = 0.05 [27], then N0 has to be increased to account for the effect of clustering by a factor of DEFF = 1+7.38×ICC = 1.37 for unit clustering. After appropriate upward rounding, this yields a total sample size of *N* = 288 service users, of whom 16 × 12 = 192 will be randomly assigned to the experimental condition (DMMH + additional implementation strategies + TAU) and 8 × 12 = 96 to the control condition (TAU). Further, based on previous research by IMMERSE partners and a meta-analysis investigating attrition in smartphone-based interventions [28], we expect an attrition of 35.5% from inclusion to 2-month post-baseline. For each of the eight clinical sites, this implies that a minimum of 54 service users will be recruited from three clusters (i.e., *n*=18 service users per cluster) and thus a total sample of 432 service users across all sites at baseline (i.e., with *n*= 16 × 18 = 288 in the experimental condition, *n* = 8 × 18 = 144 in the control condition), which allows for a 35.5% attrition rate to detect a medium effect size of d = 0.4 (with a power of 0.80, ICC=0.05, and α = 0.05). We will allow for an increase in recruitment target at all sites to up to *n*=108 per site (i.e., 36 service users per cluster) in order to compensate for delays in recruitment that may lead to overall under-recruitment across sites.

### Statistical analysis

Statistical analysis will be performed according to the intention-to-treat principle based on a pre-registration and statistical analysis plan (SAP) published on the OSF [15]. Please see the published SAP [15] for further details on the statistical analysis. The primary hypothesis of the effect of condition (i.e., DMMH + additional implementation strategies + TAU vs. TAU) on service engagement as primary outcome (measured with the patient-rated SAQ total score as the dependent variable) will be tested using a fixed effects regression model controlling for unit effects, with a dummy variable for the condition (DMMH + additional implementation strategies + TAU vs. TAU), the SAQ engagement total score at baseline, and a dummy variable coding for the random allocation to ESM outcome data collection. The focal coefficient of the model is the coefficient for the dummy variable for condition, tested via t-test for the null hypothesis of no difference between the two conditions against the two-sided alternative hypothesis that there is a difference at 2-month post-baseline. The experimental condition will be interpreted as having a statistically significant effect in the hypothesised direction if the estimated coefficient indicates a higher SAQ score (i.e., more service engagement) for this condition and the associated t-test is significant at α < .05. The primary endpoint analysis will be based on observed data using Full Information Maximum Likelihood estimation for the linear regression model. As there is minimal missing data expected in baseline and structural variables in the statistical model for the primary endpoint, we will evaluate whether more robust approaches in sensitivity analyses will be needed and would report results based on multiple imputations (please see the SAP [15] for further details).

We will test the secondary hypotheses of the effect of condition (DMMH + additional implementation strategies + TAU vs. TAU) on personal recovery, self-management, shared-decision making, personal therapy goal attainment, social functioning, social participation, quality of life, and symptom improvement/severity as secondary outcomes using a mixed model (restricted maximum likelihood estimation). First, we will use α < 0.05 to indicate statistical significance, i.e., evidence against the null hypothesis of no difference between the two conditions across all three time points (an average difference between conditions across 2-month, 6-month and 12-month post-baseline) against the two-sided alternative hypothesis that there is a difference; second, we will use, for each secondary outcome, linear contrasts at each of the three follow-up time points to investigate whether there is a potential difference between the conditions (adjusted nominal level α/3 each). The covariates included in the model in addition to the condition indicator will be the respective secondary outcome score at baseline, time (as a three-level factor), the baseline × time interaction, the condition × time interaction, and a dummy variable coding for the random allocation to ESM data collection at baseline. In addition to p-values, 95% confidence intervals for the time-specific treatment effects will be calculated. Clustering of measures within clinical units (and of repeated measures within participants) will be taken into account by allowing residuals within clinical units (and participants) to be correlated with an unstructured variance-covariance matrix. As ESM data have a multilevel structure, multiple ESM observations (level 1) will be treated as nested within time points (i.e., 2-month, 6-month, and 12-month post-baseline) (level 2) and time points will be treated as nested within participants (level 3). Additional analyses will investigate between-site effects (via condition-site interactions) and associations with unit-level characteristics. Another set of additional analyses will investigate a potential ESM method effect on primary and secondary outcomes due to ESM outcome data collection alone (based on the within-trial randomisation of participation in ESM outcome data collection).

### Economic evaluation

The economic evaluation serves the dual objective of providing information on the costs and cost structure of delivering the DMMH and other implementation strategies under different health care systems and of establishing the intervention’s value for money. As such, it will include both a micro-costing study, cost-effectiveness and cost-utility analysis conducted based on data collected as part of the cRCT. Specifically, we will adopt an activity-based approach to costing and estimate the economic costs of all key intervention activities including administration of DMMH by clinicians and delivery of implementation strategies during the implementation phase. We will use the EQ-5D-5L [29] to assess quality-adjusted life years (QALYs) and the CSRI [24] to assess use of health services, social care, informal care, and production losses as a basis for the economic evaluation. The cost-effectiveness analysis will combine cost data with patient-reported service engagement using the SAQ total score at 2-month post-baseline as the primary outcome of the cRCT. Incremental cost-effectiveness ratios (ICER) will be calculated as a measure of the incremental cost incurred by the DMMH and implementation strategies relative to their incremental benefits (based on the SAQ total score at 2-month post-baseline) in the experimental vs. control condition. Cost-utility analysis will combine cost data with quality-adjusted life years (QALYs) derived from the EQ-5D-5L and relate the incremental cost incurred by the DMMH and implementation strategies to QALYs gained at 2-month, 6-month and 12-month post-baseline in the experimental vs. control condition. In addition, we will investigate the potential cost savings which is likely generated by the expected reduction in the use of care services monitored by the CSRI [24].

### Regulatory requirements and research governance

Regulatory requirements by EU MDR 2017/745, relevant DIN EN ISO norms and IEC standards need to be addressed and overcome for successful implementation of ESM-based monitoring, reporting and feedback via the DMMH in mental health care practice. The current study is carried out as an ‘Other Clinical Investigation’ according to §82 of the EU MDR 2017/745 and its national implementation in Belgium, Germany, and Slovak Republic, relevant national legislation in Scotland and the UK, EN DIN ISO 14155 and associated DIN EN ISO norms and IEC standards. CIMH is the sponsor of this ‘Other Clinical Investigation’, which forms part of Work Package 7 (WP7) “Implementation Strategies, Processes, Outcomes and Costs” of the EU IMMERSE consortium, with the sponsor of this EU consortium being KU Leuven. The ‘Other Clinical Investigation’ has received ethics approval by IECs and, where required by national legislation, formal notification of (i.e., BfArM (Germany), or approval by (i.e., FAGG (Belgium), ŠUKL (Slovak Republic)), relevant regulatory authorities was obtained (Belgium, EUDAMED No. CIV-22-08-040547-SM01; Germany, DMIDS No. DE-22-00013961; Scotland, Ref. No. 22-WS-0125; Slovak Republic, EUDAMED No. CIV-SK-22-08-040547). Amendments to the study protocol will be submitted to the relevant IEC and regulatory authorities, then communicated to all relevant parties (DMEC, TSC, the sponsor of the clinical investigation (CIMH), funder, and collaborating centres) and will be updated in the clinical trial registry. The trial has been prospectively registered with the ISRCTN registry (ISRCTN15109760, registration date: 03/08/2022). Deviations from the study protocol are monitored across all sites and managed centrally by the sponsor of the clinical investigation (CIMH). The handling of the data will be in compliance with the European General Data Protection Regulation (GDPR) and relevant DIN EN ISO norms and IEC standards. The Therapy Designer and movisensXS (for ESM outcome data collection) platforms are hosted by movisens GmbH. The research database, analysis and compute area are hosted by the data management team at Friedrich-Alexander-University Erlangen-Nuremberg. All outcome data collected will be checked for quality on an ongoing basis, archived and integrated for analysis by the data management team. Access to the locked trial data set will be provided by the data management team to the trial statistician and investigators only after completion of data collection, checking/cleaning as well as publication of the SAP on the OSF. Further details on research governance are reported in Supplementary Material 6.

## Discussion

While the mental health field has seen a growing interest in the use of digital tools for person-centred care, uptake in routine practice remains limited. The IMMERSE effectiveness-implementation study sets out to address this challenge and, in doing so, contains several novel and unique aspects. To our knowledge, the DMMH is the first ESM-based monitoring device that has been developed and is evaluated and implemented in line with the EU MDR 2017/745 and associated DIN EN ISO norms and IEC standards. It will, thus, allow for ESM-based monitoring (via the MoMent App), reporting and feedback (via the MoMent Dashboard) in routine mental health care. As such, it reflects a step change in regulatory-compliant delivery of digital interventions and services in the mental health field and will be key in facilitating more rapid ecological translation of digital innovations to routine care [2, 30]. Further, the DMMH was developed in line with principles of human-centred design research for delivery as a technology-enabled service [12], facilitated by a firm set of technological, user- and stakeholder-centred implementation strategies. The pragmatic effectiveness-implementation trial design combined with a process and economic evaluation, which is implemented across eight sites in four European countries in line with relevant CONSORT guidelines, research governance procedures required by all academic and clinical partners, as well as national medical device legislation and regulation, will allow to endorse the methodological rigour of a cRCT, whilst providing high external validity of findings on reach, effectiveness, adoption, implementation, and maintenance. Coupled with the work carried out in the wider EU IMMERSE consortium, which aims to elucidate the diverse ethical, legal and policy challenges and practical requirements for clinical implementation of digital innovations in routine mental health care, this study will contribute to the digital transformation of mental health care in Europe and advance true person-centred care, empowering service users as active partners in their treatment, self-management, and decision-making.

### Supplementary Information

Supplementary Material 1.

Supplementary Material 2.

Supplementary Material 3.

Supplementary Material 4.

Supplementary Material 5.

Supplementary Material 6.

Supplementary Material 7.

Supplementary Material 8.

Supplementary Material 9.

Supplementary Material 10.

Supplementary Material 11.

## Data Availability

No datasets were generated or analysed during the current study.

## Abbreviations

cRCT: cluster randomized controlled trial
DMEC: Data Monitoring and Ethics Committee
DMMH: Digital Mobile Mental Health
ESM: Experience Sampling Methodology
ICER: Incremental cost-effectiveness ratios
QALYs: Quality-adjusted life years
TSC: Trial Steering Committee
TAU: Treatment as Usual
SAP: Statistical Analysis Plan
