# Supplementary material for: Strategies, processes, outcomes, and costs of implementing experience sampling-based monitoring in routine mental health care in four European countries: study protocol for the IMMERSE effectiveness-implementation study
Source: BMC Psychiatry. 2024 Jun 24;24:465. doi: 10.1186/s12888-024-05839-4 (PMC11194943; doi:10.1186/s12888-024-05839-4)
Supplement: Supplementary file 1 — Supplementary Material 1. [file 12888_2024_5839_MOESM1_ESM.docx]

**Supplementary Material 1**

Figure S1. SPIRIT figure.

|  | |  | **STUDY PERIOD** | | | | | | | |
| --- | --- | --- | --- | --- | --- | --- | --- | --- | --- | --- |
|  | | **Enrolment of units** | **Allocation** | **Enrolment of participants** | **Post-allocation** | | | | | |
| **TIMEPOINT** | | ***-t_1_*** | **0** | **0** | **t_0_** |  | **t_1_** |  | **t_2_** | **t_3_** |
| **ENROLMENT** | |  |  |  |  |  |  |  |  |  |
| Assessment of clinical units for eligibility | | X |  |  |  |  |  |  |  |  |
| Allocation of clinical units to DMMH intervention + implementation strategies + TAU (n=16) or to TAU (n=8) | | X | X |  |  |  |  |  |  |  |
| Written informed consent | |  |  | X |  |  |  |  |  |  |
| Screening checklist | |  |  | X |  |  |  |  |  |  |
| **INTERVENTIONS** | |  |  |  |  |  |  |  |  |  |
| DMMH+ Implementation strategies | |  |  |  |  |  |  |  |  |  |
| TAU | |  |  |  |  |  |  |  |  |  |
| ASSESSMENTS | |  |  |  |  |  |  |  |  |  |
| SCREENING | |  |  |  |  |  |  |  |  |  |
| MRC | |  |  | X |  |  |  |  |  |  |
| Clinical Characteristics (clinical/working diagnosis of mental disorder including comorbidity of mental disorder) | |  |  | X |  |  |  |  |  |  |
|  | Family history of mental disorder |  |  | X |  |  |  |  |  |  |
|  | TAPS |  |  | X |  |  | X |  | X | X |
|  | SITBI |  |  | X |  |  | X |  | X | X |
|  | CTQ |  |  | X |  |  |  |  |  |  |
| **PRIMARY OUTCOME** | | | | | | | | | | |
| SAQ | |  |  |  | X |  | X |  | X | X |
| **SECONDARY OUTCOMES** | | | | | | | | | | |
| Data collected in Service Users | | | | | | | | | | |
|  | QPR-15 |  |  |  | X |  | X |  | X | X |
|  | MHSEQ |  |  |  | X |  | X |  | X | X |
|  | SDM-Q-9 |  |  |  | X |  | X |  | X | X |
|  | GAS |  |  |  | X |  | X |  | X | X |
|  | SFS |  |  |  | X |  | X |  | X | X |
|  | GHQ-12 |  |  |  | X |  | X |  | X | X |
|  | MANSA |  |  |  | X |  | X |  | X | X |
|  | UCLA Loneliness Scale |  |  |  | X |  | X |  | X | X |
|  | RF |  |  |  | X |  | X |  | X | X |
|  | DERS |  |  |  | X |  | X |  | X | X |
|  | WAI |  |  |  | X |  | X |  | X | X |
|  | ESM Debriefing (in case participant uses ESM) |  |  |  | X |  | X |  | X | X |
|  | Adverse trial effects |  |  |  |  |  | X |  | X | X |
| Remote data collection | | | | | | | | | | |
|  | Completion rate, degree of DMMH usage |  |  |  |  | X | X | X | X |  |
|  | Health care practice based on the use of DMMH |  |  |  |  |  | X |  |  | X |
|  | ESM-weeks: Social Functioning, quality of life, further secondary ESM outcomes |  |  |  | X |  | X |  | X |  |
|  | Mobile sensing (device activity, message and telephone activity, steps, GPS location) during intervention |  |  |  |  | X | X | X |  |  |
| Data collected in clinicians | | | | | | | | | | |
|  | SES |  |  |  | X |  | X |  | X | X |
|  | SDM-Q-9 |  |  |  | X |  | X |  | X | X |
|  | CGI |  |  |  | X |  | X |  | X | X |
|  | WAI |  |  |  | X |  | X |  | X | X |
|  | (Severe) Adverse Events |  |  | X | X | X | X | X | X | X |
| Remote data collection using the device under investigation | | | | | | | | | | |
| Symptom exacerbation, undesired effects of treatment and | |  |  |  |  |  | X |  | X | X |
| Distress, interference, burden, and any other effects directly related to the DMMH (mHealth safety) | |  |  |  |  |  | X |  | X | X |
| Unusual activity patterns (system/privacy protection) | |  |  |  |  |  | X |  | X | X |
| *Adoption and Implementation as a part of secondary outcome measures* | | | | | | | | | | |
| Implementation fidelity rating by clinicians | |  |  |  |  | X |  | X | X |  |
| Intervention fidelity based on the use of DMMH by the service user and clinician (remotely collected) | |  |  |  |  | X |  | X |  |  |
| Maintenance as a part of secondary outcome measures | | | | | | | | | | |
| Intended and actual continuation of using the DMMH (based on App and dashboard usage data) | |  |  |  |  |  | X | X |  |  |
| *Other study parameters* | | | | | | | | | | |
| *Process evaluation in clinicians, patients and admins (but only 10 in total per country)* | | | | | | | | | | |
| MTUAS in clinicians | |  |  |  | X |  |  |  |  |  |
| ORCA in admins | |  |  |  | X |  |  |  |  |  |
| Semi-structured interviews | |  |  |  |  | X | | |  |  |
| *Economic evaluation* | | | | | | | | | | |
| Sections of the CSRI | |  |  |  | X |  | X |  | X | X |
| EQ-5D-5L | |  |  |  | X |  | X |  | X | X |
| *Optional measures* | | | | | | | | | | |
| R-GTPS | |  |  |  | X |  | X |  | X | X |
| BEAQ | |  |  |  | X |  | X |  | X | X |
| LTE | |  |  |  | X |  | X |  | X | X |
| Note: CSRI (Client Service Receipt Inventory (Chisholm *et al.*, 2000)); ORCA (Organizational Readiness to Change Assessment (Helfrich *et al.*, 2009)); MTUAS (Media and Technology Usage and Attitudes Scale (Rosen *et al.*, 2013)); SES (Service Engagement Scale (Tait *et al.*, 2002)); SDMQ-9 (Shared Decision-Making Questionnaire (Kriston *et al.*, 2010)); SAQ (Service Attachment Questionnaire (Goodwin *et al.*, 2003)); WAI (Working alliance Inventory (Munder *et al.*, 2010)); CGI (Clinical Global Impression (Guy, 1976)); QPR-15 (Questionnaire about the Process of Recovery (Neil *et al.*, 2009)); SFS (Social Functioning Scale (Birchwood *et al.*, 1990)), MRC (Medical Research Council Socio-demographic Schedule (Mallett *et al.*, 2002)); MANSA (Manchester Short Assessment of Quality of Life (Priebe *et al.* (1999)); GHQ (General Health Questionnaire (Willmott *et al.*, 2004); TAPS (Tobacco, Alcohol and Prescription Medication Scale ((McNeely *et al.*, 2016)); MHSMQ (Mental Health Self-Management Questionnaire (Coulombe *et al.*, 2015)); GAS (Goal Attainment Scaling (Turner-Stokes, 2009)); UCLA Loneliness Scale (Russell, 1996); DERS (Difficulties in Emotion Regulation Scale (Bjureberg *et al.*, 2016)); CTQ (Childhood trauma questionnaire (Bernstein et al., 2003)); STBI (Self-injurious thoughts and behaviors (Fox et al., 2020)); EQ-5D-5L ((Herdman et al., 2011)); RF (Reflective Functioning Scale (Fonagy et al., 2016)); BEAQ (experiential avoidance (Gamez et al., 2014)); LTE (List of threatening experiences (Brugha and Cragg, 1990)), R-GTPS (Revised Green Paranoid Thought Scale (Freeman et al., 2021) | | | | | | | | | | |

References

**Bernstein, D. P., Stein, J. A., Newcomb, M. D., Walker, E., Pogge, D., Ahluvalia, T., Stokes, J., Handelsman, L., Medrano, M., Desmond, D. & Zule, W.** (2003). Development and validation of a brief screening version of the Childhood Trauma Questionnaire. *Child Abuse Negl* **27**, 169-90.

**Birchwood, M., Smith, J., Cochrane, R., Wetton, S. & Copestake, S.** (1990). The Social Functioning Scale. The development and validation of a new scale of social adjustment for use in family intervention programmes with schizophrenic patients. *Br J Psychiatry* **157**, 853-9.

**Bjureberg, J., Ljotsson, B., Tull, M. T., Hedman, E., Sahlin, H., Lundh, L. G., Bjarehed, J., DiLillo, D., Messman-Moore, T., Gumpert, C. H. & Gratz, K. L.** (2016). Development and Validation of a Brief Version of the Difficulties in Emotion Regulation Scale: The DERS-16. *J Psychopathol Behav Assess* **38**, 284-296.

**Brugha, T. S. & Cragg, D.** (1990). The List of Threatening Experiences: the reliability and validity of a brief life events questionnaire. *Acta Psychiatr Scand* **82**, 77-81.

**Chisholm, D., Knapp, M. R., Knudsen, H. C., Amaddeo, F., Gaite, L. & van Wijngaarden, B.** (2000). Client Socio-Demographic and Service Receipt Inventory--European Version: development of an instrument for international research. EPSILON Study 5. European Psychiatric Services: Inputs Linked to Outcome Domains and Needs. *Br J Psychiatry Suppl*, s28-33.

**Coulombe, S., Radziszewski, S., Trépanier, S. G., Provencher, H., Roberge, P., Hudon, C., Meunier, S., Provencher, M. D. & Houle, J.** (2015). Mental health self-management questionnaire: Development and psychometric properties. *J Affect Disord* **181**, 41-9.

**Fonagy, P., Luyten, P., Moulton-Perkins, A., Lee, Y. W., Warren, F., Howard, S., Ghinai, R., Fearon, P. & Lowyck, B.** (2016). Development and Validation of a Self-Report Measure of Mentalizing: The Reflective Functioning Questionnaire. *PLoS One* **11**, e0158678.

**Fox, K. R., Harris, J. A., Wang, S. B., Millner, A. J., Deming, C. A. & Nock, M. K.** (2020). Self-Injurious Thoughts and Behaviors Interview-Revised: Development, reliability, and validity. *Psychol Assess* **32**, 677-689.

**Freeman, D., Loe, B. S., Kingdon, D., Startup, H., Molodynski, A., Rosebrock, L., Brown, P., Sheaves, B., Waite, F. & Bird, J. C.** (2021). The revised Green et al., Paranoid Thoughts Scale (R-GPTS): psychometric properties, severity ranges, and clinical cut-offs. *Psychological Medicine* **51**, 244-253.

**Gamez, W., Chmielewski, M., Kotov, R., Ruggero, C., Suzuki, N. & Watson, D.** (2014). The brief experiential avoidance questionnaire: development and initial validation. *Psychol Assess* **26**, 35-45.

**Goodwin, I., Holmes, G., Cochrane, R. & Mason, O.** (2003). The ability of adult mental health services to meet clients' attachment needs: the development and implementation of the Service Attachment Questionnaire. *Psychol Psychother* **76**, 145-61.

**Guy, W.** (1976). ECDEU assessment manual for psychopharmacology. Dept. of Health, Education, and Welfare, Public Health Service, Alcohol, Drug Abuse, and Mental Health Administration, National Institute of Mental Health, Psychopharmacology Research Branch, Division of Extramural Research Programs: Rockville, Md. : U.S.

**Helfrich, C. D., Li, Y. F., Sharp, N. D. & Sales, A. E.** (2009). Organizational readiness to change assessment (ORCA): development of an instrument based on the Promoting Action on Research in Health Services (PARIHS) framework. *Implement Sci* **4**, 38.

**Herdman, M., Gudex, C., Lloyd, A., Janssen, M., Kind, P., Parkin, D., Bonsel, G. & Badia, X.** (2011). Development and preliminary testing of the new five-level version of EQ-5D (EQ-5D-5L). *Qual Life Res* **20**, 1727-36.

**Kriston, L., Scholl, I., Hölzel, L., Simon, D., Loh, A. & Härter, M.** (2010). The 9-item Shared Decision Making Questionnaire (SDM-Q-9). Development and psychometric properties in a primary care sample. *Patient Educ Couns* **80**, 94-9.

**Mallett, R., Leff, J., Bhugra, D., Pang, D. & Zhao, J. H.** (2002). Social environment, ethnicity and schizophrenia. A case-control study. *Soc Psychiatry Psychiatr Epidemiol* **37**, 329-35.

**McNeely, J., Wu, L. T., Subramaniam, G., Sharma, G., Cathers, L. A., Svikis, D., Sleiter, L., Russell, L., Nordeck, C., Sharma, A., O'Grady, K. E., Bouk, L. B., Cushing, C., King, J., Wahle, A. & Schwartz, R. P.** (2016). Performance of the Tobacco, Alcohol, Prescription Medication, and Other Substance Use (TAPS) Tool for Substance Use Screening in Primary Care Patients. *Ann Intern Med* **165**, 690-699.

**Munder, T., Wilmers, F., Leonhart, R., Linster, H. W. & Barth, J.** (2010). Working Alliance Inventory-Short Revised (WAI-SR): psychometric properties in outpatients and inpatients. *Clin Psychol Psychother* **17**, 231-9.

**Neil, S. T., Kilbride, M., Pitt, L., Nothard, S., Welford, M., Sellwood, W. & Morrison, A. P.** (2009). The questionnaire about the process of recovery (QPR): A measurement tool developed in collaboration with service users. *Psychosis* **1**, 145-155.

**Priebe, S., Huxley, P., Knight, S. & Evans, S.** (1999). Application and results of the Manchester Short Assessment of Quality of Life (MANSA). *Int J Soc Psychiatry* **45**, 7-12.

**Rosen, L. D., Whaling, K., Carrier, L. M., Cheever, N. A. & Rokkum, J.** (2013). The Media and Technology Usage and Attitudes Scale: An empirical investigation. *Computers in human behavior* **29**, 2501-2511.

**Russell, D. W.** (1996). UCLA Loneliness Scale (Version 3): Reliability, Validity, and Factor Structure. *Journal of Personality Assessment* **66**, 20-40.

**Tait, L., Birchwood, M. & Trower, P.** (2002). A new scale (SES) to measure engagement with community mental health services. *J Ment Health* **11**, 191-8.

**Turner-Stokes, L.** (2009). Goal attainment scaling (GAS) in rehabilitation: a practical guide. *Clin Rehabil* **23**, 362-70.

**Willmott, S. A., Boardman, J. A., Henshaw, C. A. & Jones, P. W.** (2004). Understanding General Health Questionnaire (GHQ-28) score and its threshold. *Soc Psychiatry Psychiatr Epidemiol* **39**, 613-7.
