# Supplementary material for: Strategies, processes, outcomes, and costs of implementing experience sampling-based monitoring in routine mental health care in four European countries: study protocol for the IMMERSE effectiveness-implementation study
Source: BMC Psychiatry. 2024 Jun 24;24:465. doi: 10.1186/s12888-024-05839-4 (PMC11194943; doi:10.1186/s12888-024-05839-4)
Supplement: Supplementary file 2 — Supplementary Material 2. [file 12888_2024_5839_MOESM2_ESM.docx]

**Supplementary Material 2.** Study population and eligibility criteria

Service users presenting to collaborating mental health services will be approached by their treating clinician, who will provide initial information about the study. If the service user is interested in the study, the treating clinician will ask for permission to forward their contact details to the research team. If the service user agrees, the research team will contact the potential participant and provide both oral and written information of the study. After this first contact, potential participants (and, if applicable, their parents/legal guardians) will have sufficient time to decide whether they want to take part in the study. Written informed consent will then be obtained from participants (if applicable, also from parents/legal guardians), which can be withdrawn by participants at any time and without having any negative consequences for their access to standard health care. Once the informed consent form has been signed, a screening checklist will be completed to assess whether they meet inclusion and exclusion criteria to establish eligibility for the study.

Clinicians will be the treating clinicians of participating service users at the participating clinical sites. Clinicians will be approached by the research team and provided with information about the study using a specific participant information sheet that describes the study. It will be emphasized that participation is voluntary, potential participants can withdraw from the study at any time without giving any reason, and withdrawal from the study or refusal to take part will not involve any consequences for the potential participant. After discussing all relevant information, a specific informed consent for clinicians will be provided. Informed consent is only signed after all questions of the participants are answered and the procedures of the study are entirely clear.

***Inclusion criteria***

*Inclusion criteria for service users are as follows:* 1) aged 14 or older; 2) language proficiency in German, English, Slovak, or Dutch; 3) help-seeking for mental health problems and deemed sufficiently unwell to be accepted for specialist mental health treatment; 4) in contact with local inpatient, outpatient or community mental health services at the participating clinical sites; 5) ability to provide informed consent.

*Inclusion criteria for clinicians are as follows:* 1) mental health professionals providing care at the participating clinical sites, 2) being the clinician in charge of treatment for included service users in one of the clinical units at the participating clinical sites.

*Inclusion criteria for health care system administrators and managers (for the process evaluation only):* members of health care system administrators and managers in the participating clinical sites.

***Exclusion criteria***

*Exclusion criteria for service users:* 1) evidence that psychiatric symptoms are precipitated by an organic cause (incl. a diagnosis of ICD-10 F00-F09); 2) significant risk to themselves or others; 3) clinical diagnosis of intellectual disability (ICD-10 F70-79) or disorders of psychological development (ICD-10 F80-89) that are sufficiently severe to impair a person’s ability to provide informed consent; 4) medical or psychological contra-indication (as judged by the clinician in charge); 5) self-reported inability or unwillingness to use a smartphone to collect ESM data; 6) not fluent and not literate in German (Germany), Dutch (Belgium), Slovak (Slovak Republic) or English (Scotland); 7) short life expectancy/terminal illness, 8) planned discharge within 4 weeks after baseline assessment (at time of inclusion).
