# Supplementary material for: Strategies, processes, outcomes, and costs of implementing experience sampling-based monitoring in routine mental health care in four European countries: study protocol for the IMMERSE effectiveness-implementation study
Source: BMC Psychiatry. 2024 Jun 24;24:465. doi: 10.1186/s12888-024-05839-4 (PMC11194943; doi:10.1186/s12888-024-05839-4)
Supplement: Supplementary file 3 — Supplementary Material 3. [file 12888_2024_5839_MOESM3_ESM.docx]

**Supplementary Material 3.** Description of DMMH and other implementation strategies

*Core strategy for implementing ESM-based monitoring, reporting, and feedback: DMMH*

The Digital Mobile Mental Health intervention (DMMH) reflects the core strategy for implementing ESM-based monitoring, reporting, and feedback in routine mental health care and consists of (1) the MoMent App (Reininghaus *et al.*, 2023, Reininghaus and Myin-Germeys, 2023), a digital application for mobile devices using ESM to systematically monitor service users’ self-reported momentary key complaints, symptoms, mood, activities, context, and treatment goals in daily life; and (2) the MoMent Management Console that allows clinicians to (a) tailor treatment goals and questionnaires that are presented by the MoMent App jointly with the individual service user, and (b) generate reports that provide meaningful information from the self-report data using the integrated MoMent Dashboard, an interface to visualize and distil the collected data into tailored feedback to the service users and their clinicians. The MoMent App and Management Console are implemented on the Therapy Designer platform, which constitutes the backend of the DMMH technological system that is developed and maintained by the movisens GmbH (Karlsruhe, Germany), a Small and Medium-sized Enterprise (SME) and partner in the IMMERSE consortium. The DMMH will be offered to service users by the clinician in charge of their treatment (i.e., psychiatrists, psychologists, specialist mental health nurses, or other key workers) during their admission to one of the clinical units randomized to the experimental condition. The DMMH will ask service users to complete a week of ESM assessment using the MoMent App, with 8 signals per day over 7 days for the ESM core questionnaire (in addition to one morning and one evening questionnaire per day). This will be followed by a face-to-face feedback session, in which service users and clinicians access the MoMent Dashboard for visualization, first, for at least 4 weeks over an initial period of 2 months. In the remainder of this 6-month period, service users and clinicians will continue to have access to the DMMH and can use it at any point during treatment, where either the clinician or the service user consider it relevant, including any point when a clinical decision needs to be made (e.g., start of treatment, evaluation of treatment, at times when symptoms change significantly). After the end of this period, there will be a 6-month maintenance period, in which service users and clinicians still have access to the DMMH but additional implementation strategies for service users and clinicians requiring active support by the research team will be discontinued.

Service users will be asked to install the MoMent App on their smartphones, where required with the support of clinicians and/or the research team. Should they not have a suitable smartphone available, this will be provided by the research team. Researchers will then provide clinicians with an identifier linked to the service user and instruct clinicians on how to set up an account for service users on the dashboard. At the start of the assessment period, the clinician and the service user will activate the MoMent App using the identifier provided and they will also set up specific settings related to the intervention. Service users will then be invited by their treating clinician to use the MoMent App in their daily life for at least 4 weeks over an initial period of 2 months. Clinicians and service users can agree on the timing of the 4 weeks during which the MoMent App will be used. ESM-based monitoring will use a time-based design with stratified random sampling, with ESM assessments scheduled at random within set blocks of time. Specifically, on each day over an assessment period of 7 days, the MoMent App will emit 8 signals/notifications at random moments within set blocks of time. Each time the MoMent App emits a signal, participants will be asked to complete the ESM-based monitoring questionnaire. This consists of a core and several add-on modules for ESM-based monitoring, including 20 standardized ESM items to assess therapy goals, key problem areas, mood (positive and negative affect), momentary quality of life, activities, and social context in the core module as well as 10 items to be selected from the add-on modules. Questions relating to personalized therapy goals and key problem areas will allow users to define these with their clinician and, in each week, select 3 therapy goals and 3 key problem areas from their personal problem list (e.g., I am struggling with my voices, I don’t feel like doing anything), which will be inquired about as well on a 7-point Likert scale, in order to optimize personalisation of the questionnaire. ESM items will be either scored on 7-point Likert scales or will be categorical, with boxes to choose from. The daily start and end time of the questionnaire can be customized to accomplish the service user’s needs and variations in the daily schedule. Similarly, the clinicians and the service user can customize the questionnaire by integrating add-on modules they consider relevant for the treatment. The MoMent App will also provide a short morning questionnaire to assess sleep quality and sleep timing as well as an evening questionnaire to assess the progress made towards reaching the therapy goals.

The questionnaire used in the DMMH has been developed according to state-of-the-art experience sampling methodology and practice (Myin-Germeys, 2022, Myin-Germeys *et al.*, 2018, Schick *et al.*, 2023). The service user and clinicians will then receive visual feedback on the relevant patterns of association for that individual. This visualization will be two-fold: 1) for the service user: online visualisation in the MoMent App providing ongoing feedback on compliance, levels of mood, activity, and social contact; 2) for the clinician and service user: a web-based visualization on the MoMent Dashboard providing ongoing feedback on compliance, therapy goals, key problem areas, mood, momentary quality of life, activities, and social context. This can be viewed and discussed with the treating clinician in charge as well as with other clinicians, who can be given access to the dashboard by the service user. Feedback sessions of 50 minutes based on a solution-focused approach are provided throughout the use of the MoMent App for service users, who are invited to discuss the data visualization with their clinician in order to facilitate, consolidate, and expand change within sessions.

*Additional implementation strategies*

Additional implementation strategies will be delivered to facilitate the use of ESM-based monitoring, reporting and feedback in routine care via the DMMH. These strategies will be standardized across sites, as far as possible, but allow for some variation between sites and flexibility in their application to address specific requirements of clinical sites and healthcare context. Treating clinicians and service users in clinical units allocated to the experimental condition will receive the implementation strategies after random allocation of clinical units to experimental and control condition, and after completion of the baseline assessment for the duration of the initial 6-month period. After the initial 6-month period, implementation strategies for service users and clinicians requiring active support by the research team will be discontinued. The chosen implementation strategies will include:

1. Technological implementation strategies: information technology system, which adheres to prevailing standards and regulations (see section on ‘Research Governance’ below);
2. Implementation strategies for clinicians from clinical units randomized to experimental condition: an intervention manual (consistent with the Template for Intervention Description and Replication Checklist) (Hoffmann *et al.*, 2014), a manualized training, feedback (i.e., ‘key performance indicators’ on use of DMMH app/dashboard by clinicians, the number of service users seen/included, the number of sessions conducted with the service users and the duration of the sessions), and support package for clinicians and services, to facilitate the use of ESM-based monitoring, reporting and feedback via the DMMH with service users. Using the DMMH data, we will generate monthly feedback reports (with numbers on included patients and completion rates) for leading clinicians/managers, who are responsible for the implementation in order to remind and motivate them;
3. Implementation strategies for service users: a well-balanced manualized package of tailored information, counselling, and reminders for service users to motivate and enable them to use the DMMH. In each clinical site, a named individual will be available for users to address technical questions about the DMMH;
4. Organisational implementation strategies for clinical units randomized to the experimental condition: the practical implementation will be carefully prepared and planned in workshops and further contacts with leading clinicians, managers and directors at each of the sites. This will cover at least the following aspects: 1) one or two named individuals will be asked to organise the practical/logistical uptake of the DMMH in clinical practice (e.g., a research assistant); 2) the line manager/clinician who is responsible for DMMH will be identified; 3) we will also explore whether there are local clinical opinion leaders for the DMMH (through the workshops) and, if present, involve them in the communication and training activities. These strategies will purposefully vary somewhat between the different clinical sites to address local requirements.

Service users and clinicians will have access to implementation strategies for a period of 6 months, after which they will be allowed to continue to use the DMMH, but implementation strategies for service users and clinicians requiring active external support by the research team will be discontinued. This will allow for maintenance to be assessed at 6-month and 12-month post-baseline. Service contacts will be assessed for the duration of the trial using the Client Service Receipt Inventory (CSRI) (Chisholm *et al.*, 2000) to assess length of treatment and adjunct DMMH, monitor variation in delivery of, and engagement with, mental health services and digital technology.
