# Supplementary material for: Strategies, processes, outcomes, and costs of implementing experience sampling-based monitoring in routine mental health care in four European countries: study protocol for the IMMERSE effectiveness-implementation study
Source: BMC Psychiatry. 2024 Jun 24;24:465. doi: 10.1186/s12888-024-05839-4 (PMC11194943; doi:10.1186/s12888-024-05839-4)
Supplement: Supplementary file 4 — Supplementary Material 4. [file 12888_2024_5839_MOESM4_ESM.docx]

**Supplementary Material 4.** Outcome measures

After obtaining written informed consent and eligibility assessment, participants will complete a range of self-report, and clinician-rated measures to assess primary and secondary outcomes relating to **R**each, **E**ffectiveness, **A**doption, **I**mplementation, and **M**aintenance of the DMMH (based on the RE-AIM framework (Glasgow *et al.*, 1999)), covering service user experience, implementation outcomes, and mental health outcomes using the electronic Case Report Form by MaganaMed GmbH (Regensburg, Germany). All primary and secondary outcomes will be assessed at baseline (t_0_), 2-month post-baseline (t_1_), 6-month post-baseline (t_2_), and 12-month post-baseline (t_3_). All assessments will be checked on an ongoing basis for quality and completeness.

***Primary outcome***

Following the RE-AIM framework (Glasgow *et al.*, 1999), the evaluation in this implementation trial will focus on a range of outcomes relating to **R**each, **E**ffectiveness, **A**doption, **I**mplementation, and **M**aintenance of the DMMH, covering service user experience, implementation outcomes, and mental health outcomes. The primary outcome has been selected for investigating the **E**ffectiveness of implementing the DMMH.

***Effectiveness***: The **primary outcome** will be patient-reported service engagement assessed with the Service Attachment Attachment Questionnaire (SAQ) total score (Goodwin *et al.*, 2003) at 2-month post-baseline, a measure of service users’ experience of, and engagement with, their treatment and service. It captures a proximal effect of DMMH implementation on service users’ interaction with mental health services and, hence, reflects a primary indicator of implementation success.

***Secondary outcomes***

Secondary outcomes have been selected to examine **R**each, **E**ffectiveness, **A**doption, **I**mplementation, and **M**aintenance of the DMMH.

*Reach (i.e., individual-level measures of service user participation):* the number of service users consented by clinicians to offer the DMMH, the number of service users participating in, and dropping out from, the DMMH during the initial 6-month period will be recorded. A structured documentation list will be used in the clinical centres for this purpose. We will also assess DMMH usage and compliance (e.g., completion rate, degree of DMMH usage) with, as well as usability and acceptability and other metrics of the DMMH, see additional table 1.

*Effectiveness (cont.):* The following secondary outcomes will be assessed based patient- and/or clinician-rated measures with high reliability and validity at 2-month, 6-month, and 12-month post-baseline:

- clinician-rated service engagement measured with the Service Engagement Scale (SES) total (Tait *et al.*, 2002) as well as patient-reported service engagement, measured with the SAQ total score (Goodwin *et al.*, 2003) at 6- and 12-month post-baseline as a secondary outcome - both SES and SAQ capture a proximal effect of DMMH implementation on service users’ interaction with mental health services and, hence, reflect a relevant indicator of implementation success;
- personal recovery measured with the patient-rated Questionnaire about the Process of Recovery (QPR-15) total score (Neil *et al.*, 2009) - the QPR reflects a widely established measure of personal recovery used in previous mHealth monitoring studies (Gumley *et al.*, 2020);
- self-management measured with the patient-rated Mental Health Self-management Questionnaire (MHSEQ) total score (Coulombe *et al.*, 2015) - the MHSEQ is a psychometrically sound measure designed for studies on the effectiveness of self-management interventions to capture this important aspect of the DMMH (Coulombe et al., 2015);
- shared decision making measured with the patient- and clinician-rated 9-item Shared Decision-Making Questionnaire (SDM-Q-9) total score (Kriston *et al.*, 2010) to be used with the service user and treating clinician in charge, respectively - the SDM-Q-9 is a clinically relevant and short measure of shared decision making as an important secondary outcome;
- achieving personalized therapy goals measured with Goal Attainment Scaling (GAS) T score calculated using the established scoring method available in the UKROC software (Turner-Stokes, 2009) to capture this highly relevant secondary outcome;
- social functioning measured with the interpersonal functioning subscale of the patient-rated Social Functioning Scale (SFS) (Birchwood *et al.*, 1990), and with ESM (Harvey *et al.*, 2011, Schneider *et al.*, 2017) - while the SFS is a widely established measure of social functioning in the target population, ESM allows to capture experience-based social functioning of the (ESM-based) DMMH;
- mental ill-health measured with the clinician-rated Clinical Global Impression (CGI) scales (i.e., CGI severity scale) (Guy, 1976), patient-rated General Health Questionnaire (GHQ-12) total score (Gnambs and Staufenbiel, 2018), and with ESM (Myin-Germeys et al., 2018), which will allow for triangulation of this important secondary outcome;
- quality of life measured with the patient-rated Manchester Short Assessment of Quality of Life (MANSA) mean score (Priebe *et al.*, 1999) and with ESM (Myin-Germeys et al., 2018) - while the MANSA is a short and widely established measure of quality of life, ESM allows to capture experience-based quality of life as an important outcome of the ESM-based DMMH;
- loneliness and isolation measured with three items from the UCLA Loneliness Scale (Version 3)(Russell, 1996);
- mentalizing is measured with a mean score over 8 items of the Reflective Functioning Scale (RF) (Fonagy *et al.*, 2016);
- emotion regulation measured with a brief version of the Difficulties in Emotion Regulation Scale (DERS-16) (Bjureberg *et al.*, 2016).

Outcome data collected using ESM will follow the protocol from previous experience sampling studies (Myin-Germeys *et al.*, 2018). A validated and concealed procedure for randomization will be applied independently of the research team using a 1:1 ratio for allocating service users to either participation in collecting outcome data on quality of life, social functioning, and mental ill-health using ESM or no participation in ESM data collection in order to control for the potential effect of ESM data collection (given ESM forms the key part of ESM-based monitoring, reporting and feedback via the DMMH in the experimental condition). For service users allocated to participation in ESM data collection, this will include a briefing session of the ESM, in which participants will be asked to complete ESM items on a personal smartphone or, if service users do not have access to a smartphone, a study smartphone provided by the research team, for a period of 6 consecutive days in the subsequent week. During the 6-day ESM period, participants will be contacted by phone to offer advice about any potential questions about the ESM and establish their adherence to instructions provided in the briefing session. At the end of the ESM period, participants will be asked to complete a short debriefing questionnaire asking to provide feedback on the ESM and report compliance with the instructions. For participants, who are allocated to ‘no participation in ESM data collection’, baseline assessment will only include self-report, clinician-rated and researcher-rated measures.

*Adoption and Implementation:* A checklist for usage of key components of the DMMH and implementation strategies will be completed for each individual service user and clinician during the initial 6-month period to assess adoption (defined as the proportion of service users and clinicians having used a component) and implementation (defined as the extent to which a component is delivered as intended) at 2-month, 6-month, and 12-month post-baseline. Specifically, adoption will be assessed based on the proportion of clinicians and service users having used key components of the DMMH and other implementation strategies, see additional table 2. Implementation will be assessed based on collecting data on the following aspects:

- implementation fidelity: extent to which the planned implementation strategies have been used as intended by clinicians during the initial 6-month period (based on a checklist at 2-month and 6-month post-baseline)
- intervention fidelity: use of DMMH (frequency and timing of completing the DMMH (service users), delivery of the DMMH not as intended, progression toward individual treatment goals (service users)
- health care practice: number of clinical decisions made by clinicians based on the DMMH, frequency of shared clinical decisions (by service users and clinicians) based on the DMMH, frequency of changes in care in response to the DMMH, burden for clinicians to use the DMMH assessed using a self-report measure in service users and clinicians at baseline, 2-, 6-month post-baseline.

*Maintenance:* We will assess intended and actual continuation of using the DMMH (based on App and dashboard usage data) at 6-month (t_2_) post-baseline during which service users and clinicians continue to have access to the DMMH and implementation strategies and 12-month (t_3_) post-baseline during which service users and clinicians still have access to the DMMH but implementation strategies for service users (i.e., a well-balanced package of tailored information, counselling, and reminders for service users to motivate and enable them to use the DMMH) and clinicians (i.e., an intervention manual, training, feedback, and a support package for clinicians to facilitate the use of the DMMH with service users) requiring active support by the research team will be discontinued. Intended continuation of use will be assessed using two items ("How likely are you to continue using DMMH after the end of the trial?", "Would you recommend DMMH to other service users / clinicians?") , see additional table 3. An exploration of maintenance, or sustainability, will also form part of the process evaluation.

***Other measures***

Other study parameters will include basic socio-demographic characteristics based on the MRC socio-demographic schedule (Mallett *et al.*, 2002), clinical/working diagnosis of mental disorder (incl. comorbidity of mental disorders), family history of mental disorder at screening. The Working Alliance Inventory (WAI-P, WAI-T (Munder *et al.*, 2010)) will be used to assess the relationship between clinician and service user at baseline, 2-month, 6-month, and 12-month post-baseline. In addition, the Media and Technology Usage and Attitudes Scale (MTUAS; (Rosen *et al.*, 2013)) will be used to assess affinity for technology in clinicians at baseline. Detailed data will be collected to characterize clinical units by cluster-level variables.
