# Supplementary material for: Strategies, processes, outcomes, and costs of implementing experience sampling-based monitoring in routine mental health care in four European countries: study protocol for the IMMERSE effectiveness-implementation study
Source: BMC Psychiatry. 2024 Jun 24;24:465. doi: 10.1186/s12888-024-05839-4 (PMC11194943; doi:10.1186/s12888-024-05839-4)
Supplement: Supplementary file 5 — Supplementary Material 5. [file 12888_2024_5839_MOESM5_ESM.docx]

**Supplementary Material 5.** Assessment of safety

Device deficiencies, Adverse Events (AEs), and Serious Adverse Events (SAEs) will be monitored and documented throughout the study period in line with a safety manual based on the EU Medical Device Regulation (MDR 2017/745). In case of occurrence, SAEs will be reported by the sponsor of the clinical investigation (i.e., the CIMH), to the relevant regulatory authority (i.e., the Federal Agency for Medicines and Health Products (Federaal Agentschap voor Geneesmiddelen en Gezondheidsproducten (FAGG), Belgium), Federal Institute for Drugs and Medical Devices (Bundesinstitut für Arzneimittel und Medizinprodukte (BfArM), Germany), State Institute for Drug Control (Štátny ústav pre kontrolu liečiv (ŠUKL), Slovakia) or Independent Ethics Committee (IEC) (i.e., in Scotland, UK) and the Data Monitoring and Ethics Committee (DMEC). Whilst carefully documented, it is not expected that any SAE will occur as a result of the intervention. The DMEC will advise on any ethical or safety concerns, monitor evidence for intervention harm (e.g. SAEs) for the experimental condition and review whether these events are in line with expectations. If deemed necessary, the DMEC can recommend to the Coordinator and TSC for interim analyses to be conducted and the trial to be terminated prematurely. All reported (serious) adverse events which are related to the study will be reported in trial publications.
