# Supplementary material for: Strategies, processes, outcomes, and costs of implementing experience sampling-based monitoring in routine mental health care in four European countries: study protocol for the IMMERSE effectiveness-implementation study
Source: BMC Psychiatry. 2024 Jun 24;24:465. doi: 10.1186/s12888-024-05839-4 (PMC11194943; doi:10.1186/s12888-024-05839-4)
Supplement: Supplementary file 6 — Supplementary Material 6. [file 12888_2024_5839_MOESM6_ESM.docx]

**Supplementary Material 6.** Research governance

Consistent with the research governance procedures of this ‘Other Clinical Investigation’, the Chief Investigator and Sponsor Representative will have overall responsibility for the trial and will ensure implementation of sponsor responsibilities in line with DIN EN ISO 14155 as well as standard operating procedures of the sponsor (CIMH). The Trial Manager is responsible for the day-to-day management of the project. Each site appoints its lead scientist on the project as Principal Investigator (PI). The Chief Investigator, Coordinating Investigator and Trial Manager will liaise closely and meet monthly with all PIs and the research team to manage recruitment, progress, and consent procedures. It is chaired by the Chief Investigator and will manage the day-to-day running of the study, audit the trial conduct, and oversee preparation of reports to the IEC, regulatory authorities, the TSC, and the DMEC. The Chief Investigator is responsible for arranging and permitting trial-related monitoring, audits and inspections by regional and national authorities. The Chief investigator, the Coordinating Investigator, and Principal Investigators at trial sites do not have any financial or competing interests as declared in a conflict of interest statement.

The TSC will meet at least annually to provide independent overall supervision of the trial. Also, the DMEC will meet at least annually. The DMEC will advise on ethical or safety concerns and, for the experimental condition, monitor evidence for intervention harm (e.g. SAEs) and review whether these events are in line with expectations. The DMEC can recommend to the Chief Investigator and TSC to be given access to all trial data as well for interim analyses to be conducted and the trial to be terminated prematurely if deemed necessary.

Following our dissemination strategy, trial results will be published in peer-reviewed journals (open access). Lay summaries will be made available on the website of the consortium (<https://www.immerse-project.eu>).
