# Supplementary material for: Strategies, processes, outcomes, and costs of implementing experience sampling-based monitoring in routine mental health care in four European countries: study protocol for the IMMERSE effectiveness-implementation study
Source: BMC Psychiatry. 2024 Jun 24;24:465. doi: 10.1186/s12888-024-05839-4 (PMC11194943; doi:10.1186/s12888-024-05839-4)
Supplement: Supplementary file 9 — Supplementary Material 9. [file 12888_2024_5839_MOESM9_ESM.docx]

# CONSENT FORM

*IRAS ID:* 318332 *Centre Number: Study Number:*

*Participant Identification Number for this trial:*

**Title of Project:** Strategies, processes, contextual factors, outcomes, and costs of implementing Digital Mobile Mental Health in routine care in four European countries: A parallel randomized control trial

| **Short Title** | *IMMERSE* |
| --- | --- |
| **Protocol Number** |  |
| **Project Sponsor** | Central Institute of Mental Health Mannheim |
| **Coordinating Principal Investigator/**  **Principal Investigator** | *Prof Matthias Schwannauer/ University of Edinburgh, NHS Lothian*  *Dr Belinda Hacking/ Dr Claire Wallace, NHS Lothian* |
| **Name of Researcher** |  |

**Please initial each box if you agree**

| 1. I confirm that I have read the information sheet dated 26.06.23 (version 3.4) for the above study. I have had the opportunity to consider the information, ask questions and have had these answered satisfactorily. | *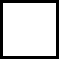* |
| --- | --- |
| 2. I understand that my participation is voluntary and that I am free to withdraw at any time without giving any reason, without my medical care or legal rights being affected. | *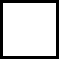* |
| ***I consent to take part in the clinical study and study procedures described herein. I understand that my participation also entails:*** | |
| 3. My name and contact details being collected during the study as described to me and be stored and accessed by researchers at the University of Edinburgh and NHS Lothian, the study sponsors, as well as relevant ethics committees, where it is relevant to my taking part in this research, or for auditing/monitoring purposes. | *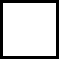* |
| 4. My pseudo-anonymised data being used by the sponsor or by people or companies or acting on its behalf or working with the sponsor for research and non-commercial use only. Such information may be shared anonymously (with Study ID and other identifiers removed). | *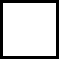* |
| 5. I understand that relevant sections of my medical notes may be looked at by individuals from the research team (NHS Trust and University of Edinburgh), as well as the study sponsors and relevant ethics committees for auditing/monitoring purposes. | *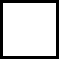* |
| 6. I agree to my General Practitioner being informed of my participation in the study. | *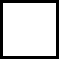* |
| 7. I understand that the information held and maintained by The University of Edinburgh may be used to help contact me or provide information about my health status. | *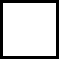* |
| 8. I consent to the MovisensXS app recording objective smartphone usage data. | *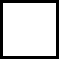* |
| 9. I consent to study appointments also taking place by means of video calling. | *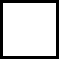* |
| 10. I consent to audio recordings being made during semi-structured interviews | *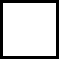* |
| 11. I consent to the Dashboard recording objective usage data | *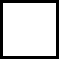* |
| 12. I consent to be contacted to participate in future research | *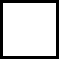* |
| 13. I agree to take part in the above study. | *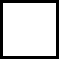* |
| 14*. I would like to see a summary of this study’s findings once the research has been completed* | *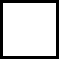* |

|  | STUDY PARTICIPANT | PARENT’S or Legally Acceptable Representative’s (LAR) |
| --- | --- | --- |
| FULL NAME (capital letters) |  |  |
| DATE  (dd-mm-Year) |  |  |
| SIGNATURE |  |  |

|  | PERSON OBTAINING CONSENT | |
| --- | --- | --- |
| FULL NAME (capital letters) |  | |
| DATE  (dd-mm-Year) |  | SIGNATURE |

# ADDITIONAL INFORMATION

## **Additional guidance on the confidentiality of your data**

| **Your** **anonymised data^^[[1]](#footnote-1)^^** | All of your data collected at the study site with your name and contact details have been replaced by a code. This is done by the local investigator who holds the link between your name/ contact details and the code to ensure your safety and confidentiality, which keeps your data anonymised.  Coded information cannot identify you unless your key worker provides your name or contact details, where allowed by applicable law. We aim to protect your data by having it safely secured and used only by the research team for analysis, and your clinician for relevant treatment. |
| --- | --- |
| **Sponsor details** | Sponsor:  Central Institute of Mental Health, Mannheim, Germany  The sponsor has the overall responsibility for a clinical study. |
| **Study site details** | Study site: *NHS Lothian*  The place where the clinical study is taking place and where you will have to go for the planned visits.  Clinical Investigator: *Dr Claire Wallace or Dr Belinda Hacking* |
| **What are the risks related to my data?** | With any research study using digital technology, there is risk of data loss, data breaches, mixing up patient data, coupling your phone with the wrong clinician, and/or lost cell phones. If data is compromised, we will document and record these adverse effects via our standard protocol. If you would like a copy of this protocol, please tell a member of the research team. |
| **If you have complaints about your data** | Study site DPO:  Dr Rena Gertz  Tel: [51 4114](tel:51%204114)  Email: [Rena.Gertz@ed.ac.uk](mailto:Rena.Gertz@ed.ac.uk)  If you are unhappy with how your data has been/is being handled, please get in touch with the research team or the Data Protection Officer to access our complaints procedure. Alternatively, you can complain to the Information Commissioner’s Office (ICO) (www.ico.org.uk or 0303 123 1113). If you wish to contact the DPO of the sponsor, please be aware that your name is not known there. You would need to link your identity to your study participant number which may compromise the coding of your data. |
| **Safeguards** | Appropriate safeguards will be implemented to protect coded data during and after the study and may include that:   - Access to the coded data will be limited to specific individuals in the local research team subject to confidentiality obligations (including the obligation to not attempt to re-identify individuals/ decode the clinical data). - The coded data will be protected with security measures to avoid data alteration, loss and unauthorised accesses and further de-identification techniques may be applied. - A data protection impact assessment (DPIA) will apply to identify and mitigate privacy risks, if any, associated with each scientific research. - When required by applicable law, scientific research is subject to the approval of Ethics Committees.   The coded data will not be shared for direct marketing purposes or other commercial purposes that are not legal duties or are not considered scientific research according to the applicable data protection legislation. |
| **Security measures: How is my data protected in other countries?** | The processing of your data starts at the study site. Your data will then be transferred to several data experts to be verified and for results to be calculated. In addition to having your data coded, your data is also protected by high standard technical security means such as strong access control and encryption. They are also protected legally by the following means:   - Within the **European Economic Area** (EEA), the data privacy laws and regulations are the same as GDPR *in your country*. The Data Protection Act 2018 is the UK’s implementation of the General Data Protection Regulation (GDPR) <https://ico.org.uk/for-organisations/guide-to-data-protection/introduction-to-dpa-2018/about-the-dpa-2018/> - Outside the EEA, those countries are recognised by the European Commission as providing an equivalent level of data protection: Andorra, Argentina, Canada, Faroe Islands, Guernsey, Israel, Isle of Man, Jersey and New Zealand:[*https://ec.europa.eu/info/law/law-topic/data-protection/data-transfers-outside-eu/adequacy-protection-personal-data-non-eu-countries_en*](https://ec.europa.eu/info/law/law-topic/data-protection/data-transfers-outside-eu/adequacy-protection-personal-data-non-eu-countries_en)*]* - In all other cases, your coded data are protected by contractual arrangements, Codes of Conduct or certifications which set the rules for personal information protection to those available in European countries (this could for instance be the case if the sponsor stores all its data with a US hosting company) or other alternatives set forth in the law. - Non-medical personnel acting on behalf of the study management and bound by professional secrecy, as well as health authorities and ethics committees, may also be given access to personal data solely for the purpose of verifying that the study is being carried out in accordance with the law and quality standards.   You may obtain further information as well as a copy of these measures by asking your clinical investigator. |
| **Legal basis** | You will participate in the clinical study only if you consent to it. If you do so, data related to your health and to the study must be collected and processed to evaluate the efficacy and the safety of the tested intervention, in accordance with legal requirements from:   - The clinical trials (i.e., the EU clinical trial regulation 536/2014 which requires the sponsor to collect and analyze such data before they are submitted to health authorities), and which requires follow-up and reporting of adverse events to the health authorities, and - *Art 6(1)(e), performance of a task in the public interest/exercise of official duty vested in the Controller by Statutory Instrument No. 557 (S76) of 1993 as amended, e.g., for education and research purposes, and* - *Any other applicable law*   *Please note that we are not relying on consent as a basis for processing under the GDPR, and our legal basis for processing is therefore distinct from the research ethics-related use of consent in this study.*  *Where special category (sensitive) personal data is being processed the additional bases from Article 9 is:*   - *Art 9(2)(j) for archiving purposes in the public interest, scientific or historical research purposes or statistical purposes*   The use of your coded data for future research will only be possible if you provide optional consent for it. Future research refers to the use of your coded data for non commercial scientific and/or health related research by researchers as well as universities, research hospitals, or deposit them in scientific or public research databases. However, they will not be combined with other information in a way that could identify you. Your participation in future research is voluntary. You are entitled to withdraw your consent for future research at any time, without giving a reason and without a negative effect on your standard of medical care. |

1. Coded data is just another term for “pseudonymised data”, which is used in the GDPR, which was preferred to render the document more readable. [↑](#footnote-ref-1)
