# Supplementary material for: Strategies, processes, outcomes, and costs of implementing experience sampling-based monitoring in routine mental health care in four European countries: study protocol for the IMMERSE effectiveness-implementation study
Source: BMC Psychiatry. 2024 Jun 24;24:465. doi: 10.1186/s12888-024-05839-4 (PMC11194943; doi:10.1186/s12888-024-05839-4)
Supplement: Supplementary file 10 — Supplementary Material 10. [file 12888_2024_5839_MOESM10_ESM.docx]

Additional files

**References**

**Birchwood, M., Smith, J., Cochrane, R., Wetton, S. & Copestake, S.** (1990). The Social Functioning Scale. The development and validation of a new scale of social adjustment for use in family intervention programmes with schizophrenic patients. *Br J Psychiatry* **157**, 853-9.

**Bjureberg, J., Ljotsson, B., Tull, M. T., Hedman, E., Sahlin, H., Lundh, L. G., Bjarehed, J., DiLillo, D., Messman-Moore, T., Gumpert, C. H. & Gratz, K. L.** (2016). Development and Validation of a Brief Version of the Difficulties in Emotion Regulation Scale: The DERS-16. *J Psychopathol Behav Assess* **38**, 284-296.

**Chisholm, D., Knapp, M. R., Knudsen, H. C., Amaddeo, F., Gaite, L. & van Wijngaarden, B.** (2000). Client Socio-Demographic and Service Receipt Inventory--European Version: development of an instrument for international research. EPSILON Study 5. European Psychiatric Services: Inputs Linked to Outcome Domains and Needs. *Br J Psychiatry Suppl*, s28-33.

**Coulombe, S., Radziszewski, S., Trépanier, S. G., Provencher, H., Roberge, P., Hudon, C., Meunier, S., Provencher, M. D. & Houle, J.** (2015). Mental health self-management questionnaire: Development and psychometric properties. *J Affect Disord* **181**, 41-9.

**Fonagy, P., Luyten, P., Moulton-Perkins, A., Lee, Y. W., Warren, F., Howard, S., Ghinai, R., Fearon, P. & Lowyck, B.** (2016). Development and Validation of a Self-Report Measure of Mentalizing: The Reflective Functioning Questionnaire. *PLoS One* **11**, e0158678.

**Glasgow, R. E., Vogt, T. M. & Boles, S. M.** (1999). Evaluating the public health impact of health promotion interventions: the RE-AIM framework. *Am J Public Health* **89**, 1322-7.

**Gnambs, T. & Staufenbiel, T.** (2018). The structure of the General Health Questionnaire (GHQ-12): two meta-analytic factor analyses. *Health Psychol Rev* **12**, 179-194.

**Goodwin, I., Holmes, G., Cochrane, R. & Mason, O.** (2003). The ability of adult mental health services to meet clients' attachment needs: the development and implementation of the Service Attachment Questionnaire. *Psychol Psychother* **76**, 145-61.

**Gumley, A., Bradstreet, S., Ainsworth, J., Allan, S., Alvarez-Jimenez, M., Beattie, L., Bell, I., Birchwood, M., Briggs, A., Bucci, S., Castagnini, E., Clark, A., Cotton, S. M., Engel, L., French, P., Lederman, R., Lewis, S., Machin, M., MacLennan, G., Matrunola, C., McLeod, H., McMeekin, N., Mihalopoulos, C., Morton, E., Norrie, J., Reilly, F., Schwannauer, M., Singh, S. P., Smith, L., Sundram, S., Thomson, D., Thompson, A., Whitehill, H., Wilson-Kay, A., Williams, C., Yung, A., Farhall, J. & Gleeson, J.** (2020). Early Signs Monitoring to Prevent Relapse in Psychosis and Promote Well-Being, Engagement, and Recovery: Protocol for a Feasibility Cluster Randomized Controlled Trial Harnessing Mobile Phone Technology Blended With Peer Support. *JMIR Res Protoc* **9**, e15058.

**Guy, W.** (1976). ECDEU assessment manual for psychopharmacology. Dept. of Health, Education, and Welfare, Public Health Service, Alcohol, Drug Abuse, and Mental Health Administration, National Institute of Mental Health, Psychopharmacology Research Branch, Division of Extramural Research Programs: Rockville, Md. : U.S.

**Harvey, P., Raykov, T., Twamley, E., Vella, L., Heaton, R. & Patterson, T.** (2011). Validating the Measurement of Real-World Functional Outcomes: Phase I Results of the VALERO Study. *The American journal of psychiatry* **168**, 1195-201.

**Hoffmann, T. C., Glasziou, P. P., Boutron, I., Milne, R., Perera, R., Moher, D., Altman, D. G., Barbour, V., Macdonald, H., Johnston, M., Lamb, S. E., Dixon-Woods, M., McCulloch, P., Wyatt, J. C., Chan, A. W. & Michie, S.** (2014). Better reporting of interventions: template for intervention description and replication (TIDieR) checklist and guide. *BMJ* **348**, g1687.

**Kriston, L., Scholl, I., Hölzel, L., Simon, D., Loh, A. & Härter, M.** (2010). The 9-item Shared Decision Making Questionnaire (SDM-Q-9). Development and psychometric properties in a primary care sample. *Patient Educ Couns* **80**, 94-9.

**Mallett, R., Leff, J., Bhugra, D., Pang, D. & Zhao, J. H.** (2002). Social environment, ethnicity and schizophrenia. A case-control study. *Soc Psychiatry Psychiatr Epidemiol* **37**, 329-35.

**Munder, T., Wilmers, F., Leonhart, R., Linster, H. W. & Barth, J.** (2010). Working Alliance Inventory-Short Revised (WAI-SR): psychometric properties in outpatients and inpatients. *Clin Psychol Psychother* **17**, 231-9.

**Myin-Germeys, I.** (2022). *The open handbook of experience sampling methodology: A step-by-step guide to designing, conducting, and analyzing ESM studies*. Center for Research on Experience Sampling and Ambulatory Methods Leuven.: Leuven.

**Myin-Germeys, I., Kasanova, Z., Vaessen, T., Vachon, H., Kirtley, O., Viechtbauer, W. & Reininghaus, U.** (2018). Experience sampling methodology in mental health research: new insights and technical developments. *World Psychiatry* **17**, 123-132.

**Neil, S. T., Kilbride, M., Pitt, L., Nothard, S., Welford, M., Sellwood, W. & Morrison, A. P.** (2009). The questionnaire about the process of recovery (QPR): A measurement tool developed in collaboration with service users. *Psychosis* **1**, 145-155.

**Priebe, S., Huxley, P., Knight, S. & Evans, S.** (1999). Application and results of the Manchester Short Assessment of Quality of Life (MANSA). *Int J Soc Psychiatry* **45**, 7-12.

**Reininghaus, U., Boehnke, J. R., Schick, A., Gugel, J., Heretik, A., Nagyova, I., Hajuk, M., Kiekens, G., Wensing, M., Schwannauer, M. & Myin-Germeys, I.** (2023). Strategies, processes, contextual factors, and outcomes of implementing Digital Mobile Mental Health in routine care in four European countries: data analysis plan for a parallel-group cluster randomized controlled trial. Open Science Framework.

**Reininghaus, U. & Myin-Germeys, I.** (2023). Mental Health Reform, Ecological Translation and the Future of Public Mental Healthcare. In *Foundations of Health Services Research: Principles, Methods, and Topics*, pp. 223-233. Springer.

**Rosen, L. D., Whaling, K., Carrier, L. M., Cheever, N. A. & Rokkum, J.** (2013). The Media and Technology Usage and Attitudes Scale: An empirical investigation. *Computers in human behavior* **29**, 2501-2511.

**Russell, D. W.** (1996). UCLA Loneliness Scale (Version 3): Reliability, Validity, and Factor Structure. *Journal of Personality Assessment* **66**, 20-40.

**Schick, A., Rauschenberg, C., Ader, L., Daemen, M., Wieland, L. M., Paetzold, I., Postma, M. R., Schulte-Strathaus, J. C. C. & Reininghaus, U.** (2023). Novel digital methods for gathering intensive time series data in mental health research: scoping review of a rapidly evolving field. *Psychol Med* **53**, 55-65.

**Schneider, M., Reininghaus, U., van Nierop, M., Janssens, M. & Myin-Germeys, I.** (2017). Does the Social Functioning Scale reflect real-life social functioning? An experience sampling study in patients with a non-affective psychotic disorder and healthy control individuals. *Psychol Med* **47**, 2777-2786.

**Tait, L., Birchwood, M. & Trower, P.** (2002). A new scale (SES) to measure engagement with community mental health services. *J Ment Health* **11**, 191-8.

**Turner-Stokes, L.** (2009). Goal attainment scaling (GAS) in rehabilitation: a practical guide. *Clin Rehabil* **23**, 362-70.
