# Supplementary material for: Strategies, processes, outcomes, and costs of implementing experience sampling-based monitoring in routine mental health care in four European countries: study protocol for the IMMERSE effectiveness-implementation study
Source: BMC Psychiatry. 2024 Jun 24;24:465. doi: 10.1186/s12888-024-05839-4 (PMC11194943; doi:10.1186/s12888-024-05839-4)
Supplement: Supplementary file 11 — Supplementary Material 11. [file 12888_2024_5839_MOESM11_ESM.docx]

| **Table 1.** *Reach* of service users (service user participation) | | | | | | |
| --- | --- | --- | --- | --- | --- | --- |
|  | **Recruitment/ number of service users consented in experimental condition** | **Interest in using the DMMH** | **Actual participation after consent/baseline** | **Number of participants dropping out from the DMMH during the intervention period** | **Usage of/ compliance with the DMMH** | **Acceptability*** |
| Strategies for ensuring *Reach* need further optimization | Less than 288 participants recruited (less than 100% of target sample size in clinical units of experimental condition) over the study period | Less than 75 % of individuals approached agree to participate / use DMMH | Less than 75% of participants initiate usage of DMMH after written consent obtained and baseline completed | A drop-out rate of more than 30% over the 2-months period for focused delivery of the DMMH (at t_1_). | Poor DMMH usage/compliance (<60% of participants participating in at least four weeks of DMMH during the 2-months period for focused delivery of the DMMH) | Low satisfaction with the DMMH (MoMent app and/or dashboard) in the debriefing questionnaire (i.e., mean satisfaction rating <3 on a 7-point scale) at t_1_  **and**  limited willingness to recommend the DMMH to others (service users / clinicians), i.e. rating <3 on a 7-point scale |
| *Reach* established | Successful recruitment of at least 288 participants recruited (i.e. 100% of target sample in clinical units of experimental condition) over the study period | ≥75 % of individuals approached about DMMH agreed to participate | ≥75% of participants initiate usage of DMMH after written consent obtained and baseline completed | A drop-out rate of ≤30% over the 2-months period for focused delivery of the DMMH. | Poor usage/compliance (≥60% of participants participating in at least four weeks of DMMH during the 2-months period for focused delivery of the DMMH) | Moderate to strong satisfaction with the DMMH (MoMent app and/or dashboard) in the debriefing questionnaire (i.e., mean satisfaction rating ≥3 on a 7-point scale) at t_1_  **or**  willingness to recommend the DMMH to others (service users / clinicians), i.e. rating ≥3 on a 7-point scale |
| Note: * Based on Reininghaus et al., 2022 | | | | | | |

| **Table 2.** Adoption and implementation of the DMMH in routine clinical care settings | | | | | | |  |
| --- | --- | --- | --- | --- | --- | --- | --- |
|  | **Adoption** | | **Implementation** | | | |  |
|  | **Adoption by service users** | **Adoption by clinicians** | **Implementation fidelity** | **Intervention fidelity** | **Health care practice** | | |
| Strategies for  adoption and implementation need further optimization | Less than 70% of service users in the experimental condition having used the MoMent app at least once per week in at least 4 weeks over the 2-months period for focused delivery of the DMMH as indicated by MoMent app usage data | Less than 70% of participating clinicians (trained in the DMMH) in the experimental condition having used the dashboard at least once per week in at least 4 weeks over the 2-months period for focused delivery of the DMMH (based on dashboard log-in data) | No use of any implementation strategy (multiple choice item) by clinicians or service users at 2-months or 6-months post-baseline | Limited usage of MoMent app  (i.e., less than 70% of service users having completed ≥30% of DMMH assessments in the MoMent app in at least 4 weeks over the 2-months period for focused delivery of the DMMH)  Delivery of the DMMH not as intended (i.e., mean rating > 3 for difficulty using the MoMent dashboard) and MoMent App use not as intended (i.e., mean rating >3 for difficulty using the MoMent app).  No/sow progress (<3) toward therapy goal rated by service user at 2-months or 6-months post-baseline | On average, <4 clinical decisions based on DMMH over 2-months period for focused delivery of the DMMH (at t_1_);  No shared decision made based on the DMMH data over 2-months period for focused delivery of DMMH (at t_1_);  No changes in healthcare practice made over 2-months period for focused delivery of DMMH (at t_1_);  High burden in the fidelity questionnaire of patients (i.e., mean rating on the items on time>3 on a 7-point scale)  High burden in the fidelity questionnaire of clinicians (i.e., mean rating on time >3 on a 7-point scale) | | |
| Adoption / implementation established | ≥70% of service users in the experimental condition having used the MoMent app at least once per week in at least 4 weeks over the 2-months period for focused delivery of the DMMH as indicated by MoMent app usage data | ≥70% of participating clinicians (trained in the DMMH) in the experimental condition having used the dashboard at least once per week in at least 4 weeks over the 2-months period for focused delivery of the DMMH (based on **dashboard log -in data**) | Use of **at least one** implementation strategy, on average, by clinicians or service users at 2-months post-baseline and/or 6-months post-baseline | Moderate to strong usage of MoMent app  (i.e., ≥70% of service users having completed ≥30% of DMMH assessments in the MoMent app in at least 4 weeks over the 2-months period for focused delivery of the DMMH)  Delivery of the DMMH as intended (i.e., mean rating ≤ 3 for difficulty using the MoMent dashboard) and MoMent App use as intended (i.e., mean rating ≤3 for difficulty using the MoMent app).  Moderate to strong progress (mean rating>3) toward treatment goal rated by patients at 2-months post-baseline and/or 6 months post-baseline | On average, ≥4 clinical decisions per service user made based on DMMH over 2-months period for focused delivery of the DMMH (at t_1_);  At least 1 shared decision made based on DMMH (i.e., >1 per clinician per service user) over 2-months period for focused delivery of the DMMH (at t_1_);  At least 1 change in healthcare practice made over 2-months period for focused delivery of DMMH (at t_1_);  Moderate burden (difficulty and time required for MoMent app/dashboard) assessed with the fidelity questionnaire for service users and clinicians (i.e., mean burden rating ≤ 3 on a 7-point scale) |  |  |

| Table 3. Maintenance of the DMMH in routine clinical care settings | | |  |
| --- | --- | --- | --- |
|  | **Intended maintenance** | **Actual/intended maintenance between t_1_ and t_2_** | **Actual/intended maintenance at t_3_** |
| Strategies for  ensuring maintenance need further optimization | Low intended use of the MoMent app or dashboard (mean rating <3 on a 7-point scale) by service users or clinicians at 2-months post-baseline **(fidelity questionnaire)** | No use of MoMent app (on average, <1 week for focused DMMH assessment) by service users at 6-months post-baseline;  No use of MoMent dashboard (on average, <1 log-in) by clinicians with any service user (based on dashboard log-in data) at 6-month post-baseline;  Intended use of the MoMent app or dashboard rated, on average, <3 on a 7-point scale by service users at 6-month post-baseline **(fidelity questionnaire)**;  Low willingness by service users (mean rating<3) to continue using the MoMent app with other clinicians;  Low intended continuation of DMMH usage by clinicians (mean rating<3) at 6-months post baseline | No use of MoMent app (on average, <1 week for focused DMMH assessment) by service users between 6-months and 12-months post-baseline;  No use of MoMent dashboard (on average, <1 log-in) by clinicians with any service user (based on dashboard log-in data) between 6-months and 12-months post-baseline;  Low willingness by service users (mean rating <3) to continue using the MoMent app at 12-month spost-baseline **(fidelity questionnaire)**;Low willingness of clinicians (<3) to continue using the dashboard at 12-month post-baseline **(fidelity questionnaire)** |
| Maintenance established | Moderate to strong intended use of the Moment app or dashboard (mean rating ≥3 on a 7-point scale) by service users or clinicians at 2-months post baseline **(fidelity questionnaire)** | Any use of MoMent app (on average, ≥1 week for focused DMMH assessment) by service users at 6-months post-baseline;  Any use of MoMent dashboard (on average, ≥1 log-in) by clinicians with any service user (based on dashboard log-in data) at 6-months post-baseline;  Intended use of the Moment app rated, on average, 3 or higher on a 7-point scale by service users at 6-months post-baseline **(fidelity questionnaire)**;  Moderate to strong willingness by service users (mean rating≥3) to continue using the MoMent app with other clinicians at 6-months post-baseline;    Moderate to strong intended continuation of DMMH by clinicians (≥3) at 6-months post baseline | Any use of MoMent app (on average, ≥1 week for focused DMMH assessment) by service users at 12-months post-baseline;  Any use of MoMent dashboard (on average, ≥1 log-in) by clinicians with any service user (based on dashboard log-in data) at 12-months post-baseline;  Moderate to strong willingness by service users (mean rating≥3) to continue using the MoMent at 12-months post-baseline **(fidelity questionnaire)**;  Moderate to strong willingness of clinicians (mean rating≥3) to continue using the dashboard at 12-months post-baseline  **(fidelity questionnaire)** |
